# Channels of Evolution: Unveiling Evolutionary Patterns in Diatom Ca^2+^ Signalling

**DOI:** 10.3390/plants13091207

**Published:** 2024-04-26

**Authors:** Eleanor A. Murphy, Friedrich H. Kleiner, Katherine E. Helliwell, Glen L. Wheeler

**Affiliations:** 1 Marine Biological Association, Plymouth PL1 2PB, UKkatherine.helliwell@mba.ac.uk (K.E.H.); 2 School of Biological Sciences, University of Bristol, Bristol BS8 1TQ, UK; 3 Department of Biosciences, University of Exeter, Exeter EX4 4QD, UK

**Keywords:** diatom, calcium signalling, ion channels, evolution

## Abstract

Diatoms are important primary producers in marine and freshwater environments, but little is known about the signalling mechanisms they use to detect changes in their environment. All eukaryotic organisms use Ca^2+^ signalling to perceive and respond to environmental stimuli, employing a range of Ca^2+^-permeable ion channels to facilitate the movement of Ca^2+^ across cellular membranes. We investigated the distribution of different families of Ca^2+^ channels in diatom genomes, with comparison to other members of the stramenopile lineage. The four-domain voltage-gated Ca^2+^ channels (Ca_v_) are present in some centric diatoms but almost completely absent in pennate diatoms, whereas single-domain voltage-gated EukCatA channels were found in all diatoms. Glutamate receptors (GLRs) and pentameric ligand-gated ion channels (pLGICs) also appear to have been lost in several pennate species. Transient receptor potential (TRP) channels are present in all diatoms, but have not undergone the significant expansion seen in brown algae. All diatom species analysed lacked the mitochondrial uniporter (MCU), a highly conserved channel type found in many eukaryotes, including several stramenopile lineages. These results highlight the unique Ca^2+^-signalling toolkit of diatoms and indicate that evolutionary gains or losses of different Ca^2+^ channels may contribute to differences in cellular-signalling mechanisms between species.

## 1. Introduction

Diatoms (Bacillariophyta) are a large divergent group of photosynthetic algae that belong to the stramenopile clade, alongside other photosynthetic (e.g., brown algae) and non-photosynthetic lineages (e.g., oomycetes). The photosynthetic stramenopiles (ochrophytes) likely acquired their plastids via the secondary endosymbiosis of a red algal cell [1,2]. Diatoms are found in marine and freshwater environments globally and act as a carbon sink and a major source of oxygen in the marine environment [3]. Under conditions where nutrients are not limited, diatoms are often the most abundant type of phytoplankton [4]. Diatoms may be split into two groups depending on whether they possess either radial symmetry (centric diatoms) or bilateral symmetry (pennate diatoms). Raphid pennate diatoms possess a raphe, one or two slits in the silica frustule that function in gliding motility. Pennate diatoms lacking a raphe are classed as araphid.

It is now estimated that the diatom clade emerged in the centric form approximately 190 Ma in the early Jurassic period, with the earliest fossil evidence of diatoms dated ~182 Ma [5,6]. Araphid pennate diatoms emerged in the late Jurassic period (~150 Ma), followed shortly by the raphid pennate diatoms in the early Cretaceous period [5]. An assessment of the origin of each centric diatom genus suggested that much of this diversification occurred during the Jurassic and Cretaceous periods, whilst genus origin of pennates extended over the Cretaceous and Cenozoic periods [5].

Diatoms inhabit diverse environments and need to be able to sense and respond to multiple different abiotic and biotic stimuli. Calcium ions (Ca^2+^) are used ubiquitously as an intracellular second messenger in eukaryotes, playing a role in numerous signal transduction pathways necessary for perceiving and responding to environmental stimuli [7,8]. Following detection of specific stimuli, Ca^2+^-permeable ion channels in the plasma membrane and/or endomembranes may be activated. The resultant increase in the concentration of Ca^2+^ in the cytosol is detected by an array of Ca^2+^-sensitive target proteins that mediate downstream signalling responses. A vast number of processes involving Ca^2+^ signalling have been demonstrated in plants, including temperature sensing, stomatal opening and responses to mechanical stimulation [9,10,11]. Similarly, Ca^2+^ signalling is involved in many complex processes in animals, with serious consequences when signalling abnormalities arise [7]. Exploration of Ca^2+^ signalling in diatoms remains limited although it is clear that diatoms employ Ca^2+^ signalling to respond to numerous stimuli, including nutrient resupply, cold stress, diatom-derived aldehydes, prolonged darkness, shear stress and hypo-osmotic stress [12,13,14,15,16].

Due to the inherent toxicity of Ca^2+^ ions, cells must maintain tight control over intracellular Ca^2+^ concentrations. This was likely the driving factor in the development of primitive Ca^2+^-signalling systems, whereby cells evolved efficient efflux mechanisms to facilitate the removal of Ca^2+^ from the cytosol [17]. The many combinations of different influx (Ca^2+^ channels) and efflux (Ca^2+^ pumps and exchangers) mechanisms result in a huge diversity in the spatial and temporal characteristics of cytosolic Ca^2+^ elevations, leading to stimulus-specific signalling, in which distinct Ca^2+^ ‘signatures’ are produced [18,19].

Ca^2+^-signalling mechanisms demonstrate increasing complexity throughout animal evolution, driven by an increasing requirement for intercellular signalling and the development of neuronal signalling to facilitate cognition, movement and development. The development of complex nervous systems has led to the expansion of several gene families including the 4-domain voltage-gated Ca^2+^ channels (Ca_v_), ionotropic glutamate receptors (iGLuRs) and pentameric ligand-gated ion channels (pLGICs)

Land plants (embryophytes) lack many of the well-characterised Ca^2+^ channel families found in animals, including Ca_v_, pLGICs, TRP, PX2R and IP_3_R channel types [20]. Many of these channel types are present in chlorophyte algae, suggesting that they have undergone extensive loss in the streptophyte lineage [21]. However, there is also a general trend of increasing diversity of Ca^2+^ channel families within land plant evolution, with many of the remaining channel types undergoing significant expansion.

Many protist lineages possess a broad diversity of Ca^2+^ channel types, including the Ca_v_, TRP and IP_3_Rs that play essential roles in animal signalling processes [22,23]. This supports the hypothesis that the major classes of Ca^2+^-permeable channels were present in ancestral eukaryotes. Most other photosynthetic eukaryotes do not appear to have undergone the large-scale losses of Ca^2+^ channel families seen in embryophytes. However, there remains significant variation in the channel types present, even between closely related taxa [23]. For example, the purinonergic P2XR receptors, a channel type predominantly associated with animals [24], are absent from most photosynthetic eukaryotes but they are present in prasinophytes (such as *Ostreococcus tauri*). Additionally, two-pore channels (TPCs) were identified in embryophytes and several stramenopile species, but were noticeably absent in rhodophytes, prasinophytes and chlorophytes.

An initial analysis of the Ca^2+^ channels found in diatom genomes revealed significant divergence between the centric *Thalassiosira pseudonana* and the pennate *Phaeodactylum tricornutum* [23]. Further species must therefore be examined in order to assess whether these trends reflect species-specific variability or represent broader trends between centric and pennate lineages. The number of available diatom genomes has recently increased significantly [25,26,27,28,29,30,31,32,33,34]. Our aim was to examine the diversity of Ca^2+^ channel types across a wider variety of diatom species to determine broad trends in their composition in comparison with other photosynthetic stramenopiles.

## 2. Results and Discussion

### 2.1. Four-Domain Voltage-Dependent Ca^2+^ Channel (Ca_v_)

The figure indicates the number of genes found in each class of Ca^2+^-permeable channel in diatom genomes (centric, araphid pennates and raphid pennates) alongside other representative eukaryotes (animals, plants/green algae and brown algae). Ca_v_, four-domain voltage-dependent Ca^2+^ channel; TPC, two-pore Ca^2+^ channel; EukCat, single-domain voltage-gated Ca^2+^ channel; GLR, glutamate receptor; pLGIC, pentameric ligand-gated ion channel; TRP, transient receptor potential channel; MscS, mechanosensitive ion channel; OSCA, reduced hyperosmolarity induced [Ca^2+^] increase channel; P2XR, purinergic P2X receptor; MCU, mitochondrial calcium uniporter; IP_3_R, inositol 1,4,5-trisphosphate receptor; ORAI, Ca^2+^-release activated Ca^2+^ channel. Presence of ion channel types in *Homo sapiens*, *Arabidopsis thaliana* and *Chlamydomonas reinhardtii* obtained from existing reviews [17,23,35,36,37]. A schematic tree is shown, displaying the currently established relationships between species [5].

Ca_v_ channels belong to the voltage-gated superfamily, alongside ion channels exhibiting selectivity for Na^+^ or K^+^. They are known to play an important role in signal transduction, especially in vertebrates, which utilise rapid Na^+^/Ca^2+^-based action potentials to transfer signals over long distances. Ca_v_ channels open in response to depolarising action potentials, allowing Ca^2+^ to enter the cytosol. Ca^2+^ therefore acts as a second messenger, coordinating the cellular response to membrane depolarisation. As such, Ca_v_ may be described as ‘signal transducers’, which are able to convert this electrical stimulus into a downstream response using Ca^2+^ as a signalling molecule [38]. In mammals, the downstream responses of Ca_v_ activation and generation of Ca^2+^ transients include neuronal synaptic transmission, initiation of muscular contraction and regulation of gene expression [38,39].

The alpha subunit of mammalian voltage-dependent Ca^2+^ channels has four distinct domains with six transmembrane segments (comprising 24 transmembrane segments in total) [40]. Mammalian Ca_v_ are separated into three distinct clades (Ca_v_1, Ca_v_2 and Ca_v_3) dependent on the type of signal transduction that they facilitate [41].

It has been suggested that Ca_v_ channels play an essential role in the signal transduction required for motility, hence they are a universal component of the Ca^2+^-signalling toolkit of metazoans as well as many other motile lineages [42]. Ca_v_ channels are noticeably absent in the land plant lineage, which may be explained by a reduced requirement for rapid Na^+^/Ca^2+^-based signalling and a lack of motility [21]. Interestingly, the green algal ancestors of the plant lineage possess several members of this channel group [20]. Algal Ca_v_ sequences have been directly linked to motility, with a flagella-localised Ca_v_ homologue in *C. reinhardtii* (CAV2) being required for the photophobic response to high light [43].

**Figure 1:**
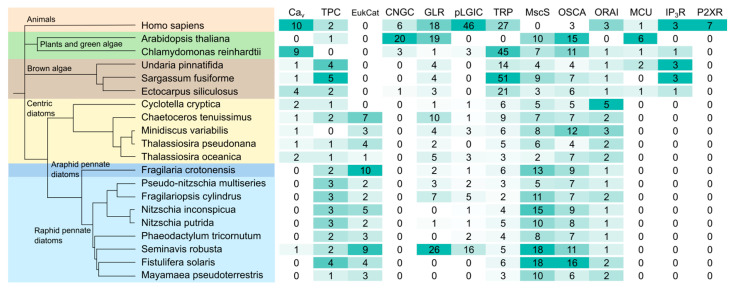
Ca^2+^ channels in diatoms and other stramenopile species.

Ca_v_ channels are present in several centric diatom species (Figure 1), supporting previous observations from transcriptomic datasets [44]. In contrast, the four-domain voltage-dependent Ca_v_ channels are absent in pennate diatoms, with the exception of *S. robusta* (Figure 1). The loss of Ca_v_ channels in pennate diatoms raises the possibility that pennates possess an alternative to these channels.

### 2.2. EukCats

In addition to Ca_v_, diatoms possess a novel class of single-domain voltage-gated Na^+^/Ca^2+^ channels, known as EukCatAs [44]. EukCatAs exhibit similarity to the prokaryote single-domain voltage-gated Na^+^ channels first identified in *Bacillus halodurans* (NaChBac) [45]. EukCat channels have also been identified in other eukaryotic phytoplankton groups, including haptophytes, dinoflagellates and cryptophytes [44], although the EukCatBs from coccolithophores are highly selective for Na^+^ [46].

We found multiple EukCatA channels in all diatom genomes, with the exception of *Cyclotella cryptica* (Figure 1). EukCatAs were not found in other stramenopiles, although some pelagophytes possess EukCatB channels [44]. The ubiquitous distribution of EukCatAs within diatoms is therefore distinct from the limited distribution of Ca_v_. EukCatAs exhibit many similar characteristics to eukaryotic Ca_v_ channels, suggesting that there may be functional redundancy between these two classes of ion channels [44,46]. The large centric diatom *Odontella sinensis* exhibits fast Na^+^/Ca^2+^-based action potentials and possesses both Ca_v_ and EukCatAs [47]. However, Ca_v_ are absent from the pennate *P. tricornutum*, and depolarisation-induced cytosolic Ca^2+^ elevations in this species are disrupted by genetic knockout of a single EukCatA orthologue [44]. Therefore, it appears that EukCatA but not Ca_v_ are essential for excitability in diatoms, at least in pennates. The apparent functional redundancy between Ca_v_ and EukCatAs may have led to the divergence of their cellular roles. Given that Ca_v_ play important signalling roles in algal flagella, it is interesting that only centric diatoms have flagellated gametes [48]. It is possible that the continued presence of four-domain Ca_v_ channels in some centric diatom genomes relates to a role in flagella motility.

### 2.3. Two-Pore Channel (TPC)

TPCs are two-domain non-selective cation channels that belong to the voltage-gated ion channel superfamily. TPCs are often localised to organellar membranes rather than the plasma membrane. TPCs in mammals are involved in many physiological processes, including membrane trafficking and autophagy regulation [49].

Animals possess three TPCs, with TPC1 and TPC2 present in all animals, while TPC3 is only present in some species. Human TPCs are localised to the endosomal and lysosomal membranes and act primarily as NAADP-activated Ca^2+^ release channels or phosphatidylinositol 3,5-bisphosphate (PI(3,5)P_2_)-activated Na^+^ channels, although they can also conduct K^+^ and H^+^ under certain conditions [50,51]. In plants, the singular TPC1 localises to the vacuolar membrane (tonoplast) and is responsible for the slow activating vacuolar (SV) current [52,53,54]. TPC1 is activated by a combination of membrane voltage and a combination of cytosolic and luminal Ca^2+^ concentrations [55]. Plant TPCs are non-selective and are primarily responsible for K^+^ transport across the vacuolar membrane. Although it was initially suggested that plant TPCs could function in Ca^2+^-induced Ca^2+^ release, a role in global plant Ca^2+^ signalling appears unlikely [55]. *A. thaliana tpc1* knockouts do not show an obvious change in phenotype [54,56]. TPCs are present in charophytes, but *Chlamydomonas reinhardtii* and other green algae do not possess a TPC [23].

**Figure 2:**
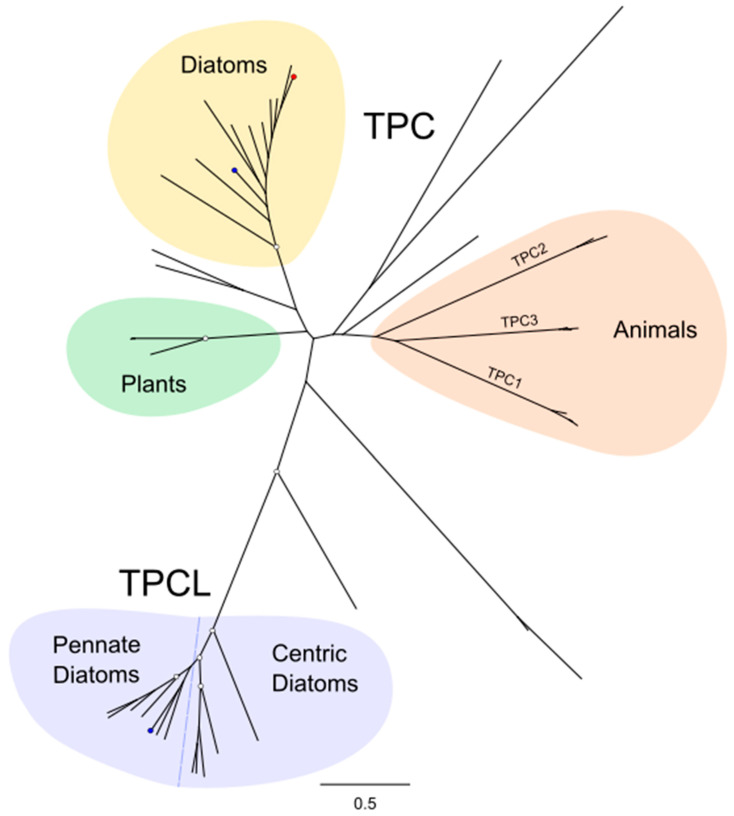
**Phylogenetic relationships between diatom two-pore channels.** The phylogenetic tree indicates the relationship between the two-pore Ca^2+^ channels of diatoms and several other eukaryotes, including plants, animals and brown algae. Diatoms possess a class of TPC (TPCL) that is distinct from other TPCs. Blue dots represent *P. tricornutum*, red dot represents *T. pseudonana*. The phylogenetic tree was generated using the maximum likelihood method with the WAG + Freqs (+F) correction model (100 bootstraps; bootstrap values > 0.7 on major nodes are shown by white circles).

TPCs were found in all diatom genomes except *M. variabilis*. The relative abundance of TPCs was greater in pennate diatoms with four different TPCs found in the pennate diatom *Fistulifera solaris*, whereas most centric species only contained a single TPC (Figure 1). Phylogenetic analysis revealed that the diatom TPCs form two distinct groups (Figure 2). Group 1 (termed TPC1) containing TPCs from both centric and pennate species forms a clade with TPCs from other eukaryotes, including animals, plants and other stramenopiles. The second group of diatom TPCs (termed TPCL) containing sequences primarily from pennate species form a separate monophyletic clade. Most pennate and centric diatoms have a single TPC1 representative, whilst pennate additionally possess 1–2 TPCL channels. TPCL channels are not restricted to pennates as a single representative is present in *C. tenuissimus* (Table 1).

**Figure 3:**
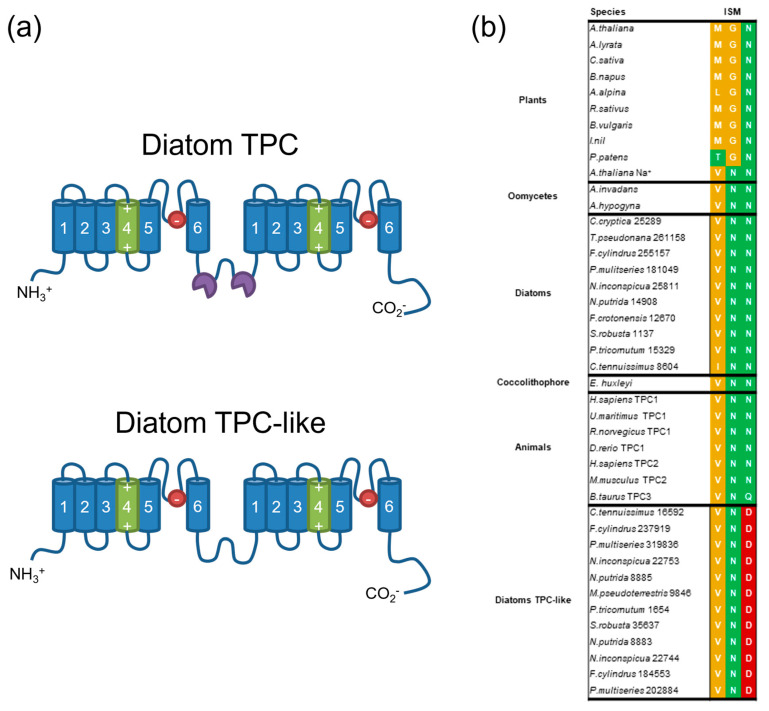
**Comparison of diatom TPC and TPCL channels.** (**a**) Schematic representation of the organism of diatom TPCs showing the two 6-transmembrane domain regions. The residues involved in voltage-gating are positioned in TM4 and the pore loops are positioned between TM5 and TM6. Diatom TPCs possess two Ca^2+^-binding EF-hands that are absent in the TPC-like (TPCL) channel. (**b**) Ion selectivity motif (ISM) from the pore loop of the second 6-TM region. The three amino acid residues in the ion selectivity motif are shown, with colours reflecting similarity in the chemistry of the amino acid side-chain. Red represents negatively charged amino acid, green represents polar uncharged amino acid, yellow represents hydrophobic amino acid. The ion selectivity motifs found in diatom TPCs are identical to those in animals, but the diatom TPCL channels have a unique motif, containing a conserved negative aspartate residue.

There has been significant debate regarding the ion selectivity of TPCs [57]. Animal TPCs show the remarkable ability to switch ion selectivity based on agonist binding. In animals, the binding of the second messenger NAADP causes TPC to become Ca^2+^-selective, whereas activation by PI(3,5)P_2_ leads to Na^+^ selectivity [58,59]. Plant TPC1 is non-selective, but can be changed to a Na^+^-selective channel if the amino acid residues in the selectivity filter of the second 6-TM region are changed to resemble human TPC2 (MGN to VNN) [57]. The selectivity filter in diatom TPCs is highly similar to animal TPCs (VNN). In contrast, the diatom TPCL channels have a unique motif incorporating a conserved aspartate residue (VND), suggesting that their ionic selectivity may differ from other TPCs (Figure 3). Animal TPC2 and plant TPC1 have two Ca^2+^-binding EF-hands positioned between the two 6-TM domains. The second EF-hand in plants is necessary for Ca^2+^-dependent channel gating. Diatom TPC1 also has two EF-hand domains, although TPCL lacks these Ca^2+^-binding domains.

**Figure 4:**
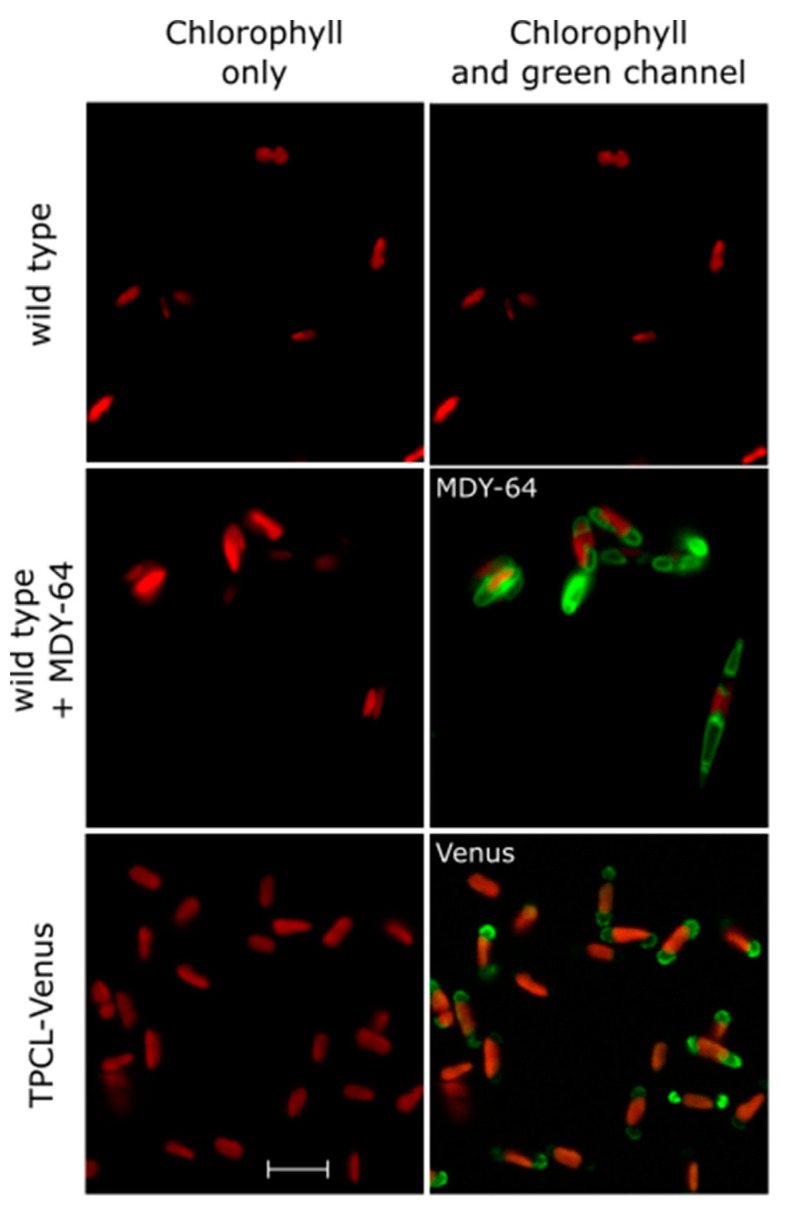
**TPCL localises to the vacuole membrane.** Confocal microscopy images of *P. tricornutum* cells: wild type (WT) (**top row**), WT treated with the fluorescent dye MDY-64 to identify the position of the vacuolar membrane (**middle row**) and cells expressing TPCL-Venus fusion protein (**bottom row**). Left column shows chlorophyll fluorescence in red. Right column shows chlorophyll fluorescence (red) superimposed with either MDY-64 or Venus fluorescence (green). Scale bar represents 10 μm. Note that the cells expressing TPCL-Venus are predominately in the oval morphotype, although the vacuoles adopt a similar position in fusiform cells (see MDY-64 image).

We examined the cellular localisation of the novel TPCL channels in diatoms. We found that *P. tricornutum* TPCL is localised to the vacuolar membrane, suggesting that it also is involved with ion fluxes across the tonoplast (Figure 4). Correct targeting of TPC1 to the tonoplast in *Arabidopsis thaliana* requires the presence of an N-terminal di-leucine motif [60]. Di-leucine motifs have also been shown to be responsible for targeting proteins to diatom vacuoles, with a C-terminal motif (EGTPLL) found in cystathione-β-synthase [61]. We identified a similar putative di-leucine motif (EASPLL) in the N-terminal region of *P. tricornutum* TPCL (position 290–295) that was highly conserved in the TPCL sequences in all pennate diatoms examined.

### 2.4. Ionotropic Glutamate Receptor-like Channel (GLR)

Ionotropic glutamate receptors (known as iGluRs in animals) are important ion channels in the transmission of neuronal signals in animals. Glutamate is a key excitatory neurotransmitter and molecular player in the processes of cognition, learning and memory [62,63]. GLRs have expanded hugely across eukaryotes, with at least 20 different families in vertebrates [64]. Animal iGLuRs have evolved to meet the increasingly complex role they play in nervous system signalling, diverging greatly in terms of protein structure and ligand specificity. They are primarily separated into four distinct clades, categorised by the agonist that activates them, although a recent analysis including a greater diversity of animal lineages indicated additional complexity in the classification of animal iGluRs [65].

Plant homologues of animal iGluRs known as glutamate receptor-like channels (GLRs) were identified in 1998 [66]. GLRs are involved in a broad range of developmental processes in plants including seed germination, root development and pollen tube growth [67,68,69,70]. They have also been implicated in responses to wounding, pathogens and various abiotic stresses [66,71]. *A. thaliana* GLRs are localised to many different cellular membranes, including the plasma membrane, chloroplast membrane, mitochondrial membrane and vacuolar membrane [67,72,73,74,75,76,77,78]. This further emphasises the broad range of functions that GLR-mediated signalling contribute to.

Vertebrates and embryophytes both possess an expanded group of iGluRs or GLRs respectively. The complement of GLRs is much smaller in many other eukaryotes. The green alga *C. reinhardtii* possesses only one GLR, indicating that the expansion of GLRs in land plants occurred after the divergence of streptophytes and chlorophytes (Edel 2017). Ionotropic GLR channels were identified in all centric diatom species but appear to absent from some pennate diatoms such as *P. tricornutum* and *F. solaris*. There is considerable variability in the number of GLRs present across diatom genomes, with substantial expansion of the GLRs found in the centric *C. tenuissimis* and the pennate *S. robusta*. We found three or four GLRs within brown algal species.

### 2.5. Pentameric Ligand-Gated Ion Channel (pLGIC)

The pLGICs are found in both a cys-loop and a cys-less form, dependent on whether they possess a 13-residue loop bounded by two cysteine residues [79]. pLGICs are thought to have particularly early origins, with the presence of orthologues in primitive groups such as cyanobacteria and proteobacteria [80]. The cys-loop pLGIC family of neurotransmitter receptors is ubiquitous in vertebrates and has important functions in the nervous system [81]. Examples include nicotinic acetylcholine receptors and γ-aminobutyric acid (GABA) receptors. Cys-less pLGICs were identified in many unicellular eukaryote groups, including diatoms (*P. tricornutum* and *Thalassiosira oceanica*), coccolithophores and chlorophytes [82]. Our analysis indicated the wider presence of cys-less pLGICs across the diatom clade, although they were absent in some species (e.g., *T. pseudonana* and *F. solaris*) (Figure 1). pLGICs were substantially expanded with the *S. robusta* genome.

In contrast to the diatoms, both the cys-loop and cys-less pLGICs were absent in the brown algal species included in this analysis. The absence of pLGICs in many stramenopile and other unicellular algae species suggests that these receptors have become functionally redundant and therefore were slowly lost over time [82].

### 2.6. Transient Receptor Potential Channel (TRP)

TRP channels are nonselective cation channels found on the plasma membrane that facilitate the transport of Na^+^ and Ca^2+^ into the cytoplasm and have an important role in sensing environmental signals and coordinating physiological responses [83]. The TRP family is greatly expanded in mammals, with six key subfamilies dividing up the 28 channels based on sequence homology [83,84]. The activation mechanism of mammalian TRP channels is diverse, with some channels being voltage-gated and others being activated by mechanosensation, temperature or ligand-binding [85]. Once activated, TRP channels cause cell membrane depolarisation and transient elevation of intracellular Ca^2+^.

Surprisingly, whilst TRPs are absent in land plants, many putative TRP channels have been identified in green algae such as *C. reinhardtii* and *Volvox carteri* [37]. Very few studies have characterised TRPs outside of the animal lineage, but *C. reinhardtii* TRP1 has been classified in a novel subfamily, which is distinct from the existing subfamilies present in mammals [37]. Many *C. reinhardtii* TRPs are localised to the flagella, such as TRP11 which is a mechanosensitive channel necessary for the flagellar-mediated avoidance response [86].

**Figure 5:**
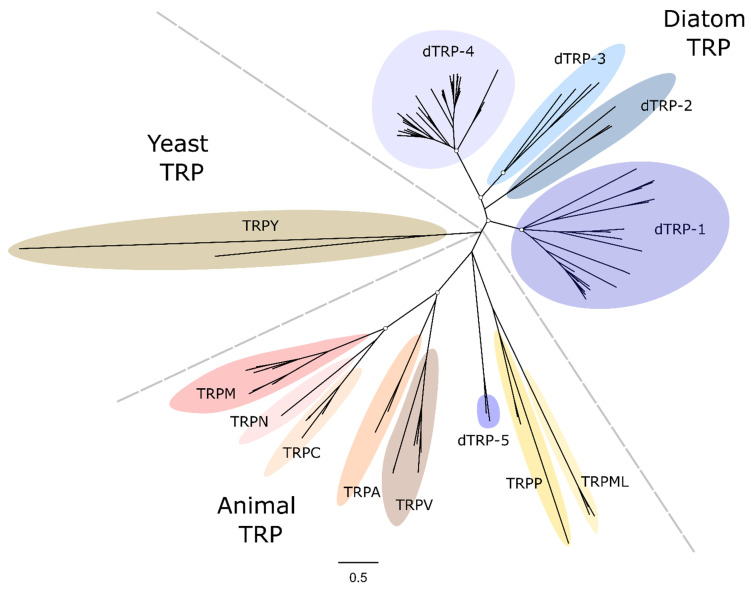
**Phylogenetic tree examining the relationships between diatom transient receptor potential (TRP) channels.** TRP channels were identified in centric diatoms (*Cyclotella cryptica*, *Chaetoceros tenuissimus*, *Thalassiosira pseudonana*, *Thalassiosira oceanica*), araphid pennate (*Fragilaria crotonensis*) and raphid pennate (*Pseudo-nitzschia multiseries*, *Fragilariopsis cylindrus*, *Nitzschia inconspicua*, *Nitzschia putrida*, *Phaeodactylum tricornutum*, *Seminavis robusta*, *Fistulifera solaris* and *Mayamaea pseudoterrestris*). For comparison, TRP channels from animal (*Homo sapiens* and *Drosophila melanogaster*) and yeast (*Rhodosporidium toruloides* and *Metschnikowia pulcherrima*) were included in the analysis. The phylogenetic tree was generated using the maximum likelihood method with the WAG + Freqs (+F) correction model (100 bootstraps; bootstrap values > 0.7 on major nodes are shown by white circles).

TRP channels were found in all stramenopile genomes examined. The TRP channel family is greatly expanded in the brown algae, particularly in *S. fusiforme* which possesses 51 TRP channels. TRP channels were found in all diatom genomes, although gene copy numbers were smaller than brown algae with only three to nine TRP channels per species, suggesting that there has not been a substantial expansion of diatom TRP channels (Figure 1). Diatom TRP channels group into four major clades that are distinct from the different classes of animal TRP channels (Figure 5). A fifth clade (dTRP-5) contains TRP sequences from just two diatom species (*C. tenuissimus* 7400 and *N. putrida* 13388) that appear to be more closely related to animal TRP channels.

### 2.7. Mechanosensitive Channel Small Conductance (MscS)

It is important for many organisms to be capable of detecting and responding to mechanical stimuli including physical pressure from touch, gravity and osmotic stress. Mechanosensitive (MS) channels are gated by membrane tension and initiate responses to these stimuli. Prokaryotes have a large and well-characterised MS ion channel family [87]. The two major groups of MS channels in prokaryotes are the mechanosensitive channel of small conductance (MscS) and large conductance (MscL) channels, characterised by their structural differences [87].

MscS homologues are found in plants, photosynthetic protists and fungi, but they are absent in animals. Plant MscS are generally nonselective and stretch-activated. One class of plant MscS is known to be activated by osmotic stress, but research is ongoing into the wider role of these channels [88,89]. Plant MscS are localised to either the plasma membrane or various organellar membranes. A MscS channel in *C. reinhardtii* (MSC1) is involved in the organisation of the chloroplast [90].

Every stramenopile species analysed within this study possesses at least two MscS channels, with the greatest numbers of MscS channels found in pennate diatoms. Members of the Naviculaceae lineage possess up to 18 MscS homologues (Figure 1). Considering the dynamic environment of many stramenopile species, MscS are likely to play an important role in diatom responses to environmental stimuli. It will be interesting to determine whether MscS channels also localise to diatom plastids and play a role in the organisation of the chloroplasts derived from secondary endosymbiosis.

### 2.8. Reduced Hyperosmolarity-Induced [Ca^2+^] Increase Channel (OSCA)

OSCA channels were first identified as Ca^2+^ channels that play an important role in the short-term responses of plants to hyperosmotic stress [91]. OSCAs are related to the TMEM63 family of ion channels found in animals. Together, the OSCA/TMEM63 family forms the largest family of mechanosensitive channels in eukaryotes [92]. In animals, these channels are critical in environmental sensing and coordination of movement. Knockout of TMEM63B in mice led to impaired hearing, due to the role of TMEM63B in the Ca^2+^-signalling pathway that modulates hair cell morphology [93]. OSCA channels are found in divergent eukaryote lineages. Four primary clades of OSCAs are found in land plants [17], with the OSCA1 clade proposed to have played an important role in enabling land plants to sense water stress as they colonise terrestrial environments [94].

**Figure 6:**
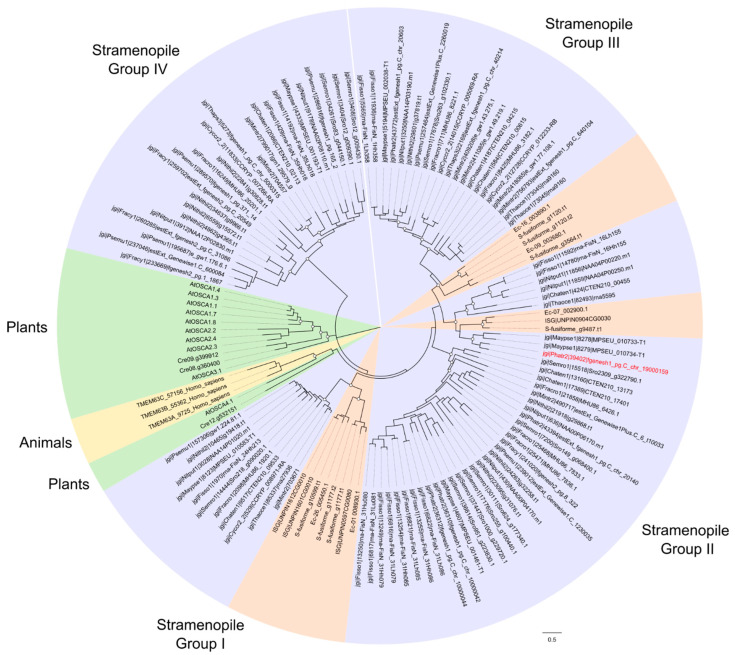
**Phylogenetic tree comparing the relationships between OSCA channels of diatoms.** Sequences were taken from the centric diatoms *Cyclotella cryptica* (Cc), *Chaetoceros tenuissimus* (Ct), *Thalassiosira pseudonana* (Tp), *Thalassiosira oceanica* (To), the araphid pennate diatom *Fragilaria crotonensis* (Fcr) and the raphid pennate diatoms *Pseudo-nitzschia multiseries* (Pm), *Fragilariopsis cylindrus* (Fcy), *Nitzschia inconspicua* (Ni), *Nitzschia putrida* (Np), *Phaeodactylum tricornutum* (Pt), *Seminavis robusta* (Sr), *Fistulifera solaris* (Fs) and *Mayamaea pseudoterrestris* (Mp). Additional OSCA/TMEM63 sequences were taken from brown algae, animals and plants. Animal species is *Homo sapiens*. Plant and green algal species are *Arabidopsis thaliana* (At) and *Chlamydomonas reinhardtii* (Cre). Brown algal species are *Undaria pinnatifida* (UNPIN), *Sargassum fusiforme* (S-fusiforme) and *Ectocarpus siliculosus* (Ec). Within the stramenopile groups, purple indicates diatoms and brown represents brown algae. The phylogenetic tree was generated using the maximum likelihood method with the WAG + Freqs (+F) correction model (100 bootstraps; bootstrap values > 0.7 on major nodes are shown by white circles).

OSCA channels were identified in all diatom species within this survey, suggesting an essential role in diatom physiology (Figure 1). Diatom OSCA sequences were distinct from the four main OSCA groups found in land plants. Instead, diatom OSCA sequences were grouped into four distinct clades, alongside sequences from brown algae (Figure 6).

**Figure 7:**
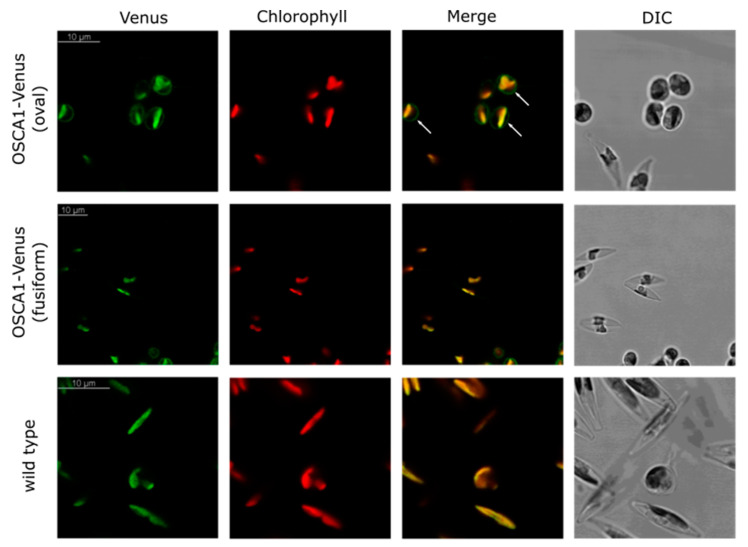
***P. tricornutum* OSCA1 localises to the plasma membrane in oval cells.** Confocal microscopy images of *P. tricornutum* oval and fusiform cells: left column shows green fluorescence to indicate position of OSCA1-Venus. Middle column shows chlorophyll fluorescence in red. Note that the OSCA1-Venus construct was under the control of its native promoter. Due to low expression levels of OSCA1-Venus, there is chlorophyll autofluorescence visible in the green channel, although the green fluorescence in the plasma membrane is clearly distinct in the merge image (arrows). Right column shows differential interference contrast (DIC) brightfield images. The green fluorescence in the plasma membrane is absent from fusiform cells (middle row) and all wild-type (WT) cells (bottom row).

The function of OSCA channels within diatoms remain unknown, but evidence shows that *OSCA1* (Pt39402) has higher expression levels in oval *P. tricornutum* morphotype cells compared to fusiform and triradiate morphotypes [95]. The oval morphotype is most likely an adaptation to benthic environments, whereas the fusiform and triradiate forms predominate in planktonic environments [96]. Expression of an OSCA1-Venus fusion in *P. tricornutum* under its native promoter indicated a plasma membrane localisation in oval cells, although no expression was detected in fusiform cells (Figure 7). Previous research has indicated that genes involved in responses to hyperosmotic and cold stress are upregulated in the oval morphotype of *P. tricornutum*, suggesting that oval cells are more resilient to environmental stresses [97]. We also recently demonstrated that oval *P. tricornutum* cells are less sensitive to hypo-osmotic stress than fusiform cells [98]. The differential expression of OSCA1 between oval and fusiform morphotypes suggests that it could contribute to these different sensitivities, although it remains to be determined whether OSCA1 plays a direct role in osmotic Ca^2+^ signalling in *P. tricornutum*.

### 2.9. P2X Purinoreceptor (P2XR)

P2X receptors are Ca^2+^-permeable ion channels that play important roles in sensing extracellular ATP in vertebrates. The phylogenetic distribution of P2X receptors is limited mainly to vertebrates due to their role in the nervous system and muscular control, although P2X receptors have been found in some algal species such as the prasinophyte *Ostreococcus tauri* [24,99]. We did not find P2X receptors in any diatom genome or the other stramenopiles assessed within this survey (Figure 1).

### 2.10. Mitochondrial Uniporter (MCU)

An important element of Ca^2+^-dependent signalling is the efflux of Ca^2+^ from the cytosol following a transient signalling event. Mitochondria are known to take up significant amounts of Ca^2+^ and thus help to regulate cytosolic Ca^2+^ concentrations [100]. The mitochondrial uniporter is a protein located on the inner mitochondrial membrane, which facilitates movement of Ca^2+^ into the mitochondrial matrix [101]. It is now considered to be part of a wider mitochondrial uniporter complex, which comprises all the components required for mitochondrial Ca^2+^ uptake [102]. The complex includes the important regulatory protein mitochondrial Ca^2+^ uptake 1 (MICU1).

Whilst there is only one member of the MCU family in mammals, six MCU homologues have been identified in A. thaliana, with MCU1 and MCU2 confirmed to be Ca^2+^-selective 36. MCU homologues are found across almost every major eukaryotic group, including other protist lineages [103]. Despite this, MCUs have a patchy presence across several different lineages, suggesting that they have been lost independently by numerous different eukaryotic groups. For example, MCUs are present in the major fungal groups Ascomycota and Basidiomycota species, but absent in the Saccharomycetales and also absent in apicomplexans and microsporidia [104]. MCUs were identified in the brown algae *E. siliculosus* and *U. pinnatifida*, which supports the existing evidence that MCUs are present in many stramenopiles (Figure 1) [104]. However, they were absent in diatoms and the brown algae *S. fusiforme* (Figure 1). Whilst some of the eukaryote lineages that have lost MCU exhibit streamlined mitochondrial metabolism (e.g., microsporidians), many others do not. It is not clear whether these lineages (including diatoms) are able to use an alternative mechanism to facilitate mitochondrial Ca^2+^ uptake.

### 2.11. Inositol 1,4,5-Triphosphate Receptor (IP_3_R)

IP_3_Rs are involved in the release of Ca^2+^ from stores in the endoplasmic reticulum (ER) in response to the intracellular messenger IP_3_, and they initiate store-operated Ca^2+^ entry (SOCE) following depletion of Ca^2+^ within the ER [105,106]. In animals, the binding of IP_3_ allows IP_3_Rs to bind Ca^2+^, resulting in the opening of the channel and the release of ER Ca^2+^ into the cytosol. The transient increase in Ca^2+^ in the cytosol and the depletion of ER Ca^2+^ stores initiate the interaction of stromal interaction molecule 1 (STIM1) with the plasma membrane-localised Orai channel. Animal IP_3_Rs are located primarily in the ER membrane and the Golgi membrane, but also can be found in the nuclear envelope [106].

IP_3_Rs are ubiquitous in the metazoans and are integral to the generation of fast Ca^2+^ waves in animal cells. They have also been identified in parasitic protozoa, such as *Trypanosoma* and *Leishmania* [107]. IP_3_Rs are absent in plants, but present in green algae such as *C. reinhardtii*, which suggests that these channels were lost relatively recently in plant evolutionary history.

Until recently, IP_3_Rs had been identified in only one algal group (the green algae). However, it is clear that IP_3_Rs are present in brown algae, as all species within this study possessed at least one IP_3_R. This supports the existing evidence of transient Ca^2+^ signals in *Fucus serratus* following treatment with IP_3_ [108]. In contrast, no IP_3_Rs were identified in the diatom lineage (Figure 1). Diatom Ca^2+^ elevations induced by hypo-osmotic stress progress as rapid Ca^2+^ waves with similar properties to those seen in metazoans [98]. In the absence of IP_3_Rs, diatom Ca^2+^ waves may be mediated by a novel channel type.

### 2.12. Ca^2+^Release-Activated Ca^2+^ Channel (Orai)

The Orai channels are the predominant type of Ca^2+^ release-activated Ca^2+^ (CRAC) channels involved in store-operated Ca^2+^ entry (SOCE), a pathway whereby a decrease in the Ca^2+^ concentration in the ER stimulates the influx of extracellular Ca^2+^ into the cytosol [109,110]. Orai channels are found in all mammalian cells and are thought to be an essential component of animal Ca^2+^-signalling pathways. Orai channels have also been identified in some photosynthetic protists and most plant groups prior to angiosperms [21]. However, this channel type is not present in angiosperms and has not been identified in fungal species including *Saccharomyces cerevisiae* [111]. Indeed, Orai channels are thought to be the most recent Ca^2+^ channel type to have been lost in the land plant lineage [111].

**Figure 8:**
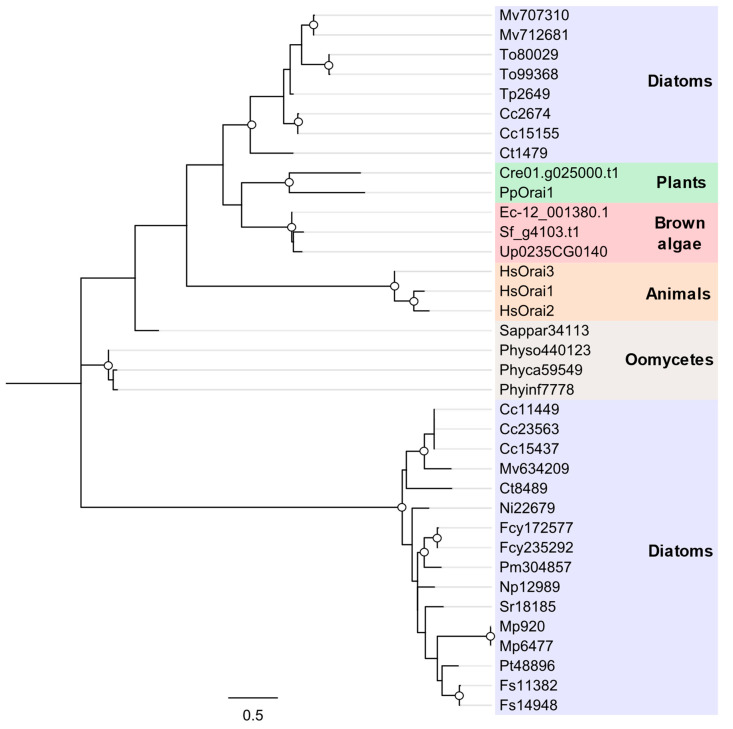
**Phylogenetic relationship between the Orai channels of diatoms and other eukaryotes.** Species are *Homo sapiens* (Hs); the plant *Physcomitrella patens (Pp)*; green alga *Chlamydomonas reinhardtii* (Cre); the brown algae *Undaria pinnatifida* (Up), *Sargassum fusiforme* (Sf) and *Ectocarpus siliculosus* (Ec); the oomycetes Saprolegnia parasitica (Sappar), *Phytophthora capsica* (Phyca), *Phytophthora sojae* (Physo) and *Phytophthora infestans* (Phyinf); the centric diatoms *Cyclotella cryptica* (Cc), *Chaetoceros tenuissimus* (Ct), *Minidiscus variabilis* (Mv), *Thalassiosira pseudonana* (Tp) and *Thalassiosira oceanica* (To); the araphid pennate diatom *Fragilaria crotonensis* (Fcr); and the raphid pennate diatoms *Pseudo-nitzschia multiseries* (Pm), *Fragilariopsis cylindrus* (Fcy), *Nitzschia inconspicua* (Ni), *Nitzschia putrida* (Np), *Phaeodactylum tricornutum* (Pt), *Seminavis robusta* (Sr), *Fistulifera solaris* (Fs) and *Mayamaea pseudoterrestris* (Mp). The unrooted phylogenetic tree was generated using the maximum likelihood method with the WAG + Freqs (+F) correction model (100 bootstraps; bootstrap values > 0.7 on major nodes are shown by white circles).

A previous study examining the distribution of the Orai channels identified homologues in the genomes of brown algae and centric diatoms, but not in pennate diatoms [21]. We identified Orai homologues in brown algae and centric diatoms that exhibited similarity to the Orai channels from plants and green algae. However, we also found a divergent group of Orai channels in diatoms that was present in both pennates and centrics (Figure 8). Several centric species appear to have both forms of Orai (e.g., *T. pseudonana*), whereas pennates only have the second group. The divergent class of Orai channels forms a distinct monophyletic clade, but retains the conserved glutamate corresponding to E178 in *Drosophila melanogaster* Orai1 that is important for Ca^2+^ selectivity.

The activation of Orai channels in animals is stimulated through the binding of stromal interaction molecule 1 (STIM1), alongside other ER Ca^2+^ sensors. The activation of Orai channels is required to initiate SOCE, therefore it is expected that organisms possessing Orai channels must also have an associated Ca^2+^ sensor. Indeed, STIM1 homologues are universally present in the animal lineage. Outside of animals and choanoflagellates, several groups known to possess an Orai channel including early plants and green algae are lacking a STIM1 homologue. Similarly, STIM1 has not been detected in diatoms. As the activation of Orai channels is required to initiate SOCE, it is likely that these groups possess a different protein that performs a similar function.

## 3. Materials and Methods

### 3.1. Bioinformatic Searches for Ca^2+^ Channels

Sequence similarity searches were used to identify candidate Ca^2+^ channel genes in diatom genomes (Supplementary Table S1). Query protein sequences for Cav, TPC, EukCat, GLR, TRP, CNGC and MscS channels were obtained for *P. tricornutum* and *T. pseudonana* from previous analyses [23]. For the Ca^2+^ channel families not previously found in these species, sequences were obtained from other protists or plants. Predicted proteins for each diatom species were downloaded from the Joint Genome Institute Phycocosm repository (https://phycocosm.jgi.doe.gov/phycocosm/home, accessed on 1 December 2022), and similarity searches were performed using BLASTP within Geneious Prime. All instances of protein absence were confirmed using TBLASTN against the genome with additional searches using sequences from closely related organisms. Proteins recovered from sequence-similarity searches were identified using a combination of BLAST score, manual inspection of conserved residues in multiple sequence alignments and their position in phylogenetic trees generated by both neighbour-joining and maximum likelihood method within the Geneious Prime (version 2024.0.5) software package. Proteins exhibiting strong hits for each channel type were then analysed using InterPro (https://www.ebi.ac.uk/interpro/, accessed on 3 March 2024) to check for the presence of conserved functional domains.

### 3.2. Phylogenetic Analyses

For detailed phylogenetic analysis of TRP, OSCA and ORAI channels, multiple sequence alignments were generated using MUSCLE within Geneious Prime. Incomplete sequences or poorly aligned regions were removed following manual inspection. Maximum likelihood phylogenetic trees were generated using MEGA11 software (version 11.0.13) with the best substitution model (WAG with frequency). The phylogenetic analysis only used sites that were present in at least 95% of all sequences within the alignment. A total of 100 bootstraps were applied. Initial unrefined alignments are provided in Supplementary Information (Supplementary Data S1–S4).

### 3.3. Localisation of TPCL and OSCA1 in P. tricornutum

For *P. tricornutum TPCL* (Protein ID 1654), the entire open reading frame with exclusion of the stop codon was amplified by polymerase chain reaction using the proof-reading Phusion polymerase (Thermo Fisher Scientific, Waltham, MA, USA) and the following primers:

PtTPCL_For ATGAGCTCGCCACGCC

PtTPCL_Rev ATTTGCGACGGGAGCAGC

The amplicon was cloned into the *StuI* sites of the pPha-TI_Venus vector using blunt end cloning (Supplementary Data S5). Expression of *TPCL* was driven by a constitutive *fcpA* promoter.

The *P. tricornutum OSCA1* gene (Protein ID 39402) including its upstream promoter region (800 bp 5′ to the translation initiation site) but excluding the stop codon was amplified using the Phire Plant Direct PCR kit (Thermo Fisher Scientific) and the following primers:

PtOSCA1_For atagaaccagatcccccgggctgcaGACCAGTGGTGGCCTTCCTC

PtOSCA1_Rev gttcttctcccttggaaaccataggAACGAAACGACGCTTGTCCG

The amplicon was cloned into pPha-TI_Venus vector via the *StuI* and *PstI* sites using a HiFi DNA Assembly Cloning Kit (New England Biolabs, Ipswich, MA, USA).

The pPha-T1_TPCL_Venus and pPha-T1_OSCA1_Venus plasmids were introduced into *P. tricornutum* wild type strain (PLY670) via biolistics using the transformation protocol described previously [44]. Cells were grown on selection plates (50% seawater supplemented with f/2 nutrients and 75 µg mL^−1^ Zeocin) to identify transformed cells. Zeocin-resistant colonies were screened for fluorescence using confocal microscopy. Cells expressing TPCL were imaged using a Zeiss LSM510 confocal microscope with a Plan Neofluar 40× oil objective (Zeiss, Oberkochen, Germany). Cells were excited with a 488 nm argon/krypton laser, and emission was detected between 500 and 530 nm for Venus and 650 and 710 nm for chlorophyll. Cells expressing OSCA1 were imaged using a Leica SP8 confocal microscope with a 63x oil objective (Leica Microsystems, Milton Keynes, UK). Cells were excited with a 488 nm argon/krypton laser, and emission was detected between 500 and 530 nm for Venus and 650 and 710 nm for chlorophyll.

## 4. Conclusions

Our analysis demonstrates a broad diversity in the types of Ca^2+^ channel present in diatoms. Several classes of ion channels that play important roles in animal Ca^2+^ signalling appear to be completely absent from diatoms, most notably MCU and IP_3_R. As these are both present in brown algae, they were most likely lost in stramenopile evolution following the divergence of the bacillariophyte and phaeophyte lineages. There is no evidence for the broad expansion of any particular Ca^2+^ channel classes across diatoms in a manner seen for GLRs and CNGCs in land plants, but we found evidence for the emergence of highly divergent forms of ion channels within diatoms, most notably within the TPC and Orai classes. Our analyses also demonstrate that diatoms possess unique Ca^2+^ channels, with the single-domain EukCatA channels present in all diatoms (except *C. cryptica*). Within the diatoms, we found evidence for some differentiation between the centric and pennate lineages. For example, the divergent form of TPC (TPCL) was predominately found in pennates and the divergent AlgalOrai was also restricted to pennates. Importantly, the four-domain voltage-gated Ca_v_ channels appear to have been lost almost entirely from pennates, with the exception of a single homologue found in *S. robusta*.

We found evidence for significant expansion of several ion channel types, including the iGluR and the pLGIC in some individual species, particularly *Seminavis robusta*. The *S. robusta* genome was recently found to possess the largest number of protein-coding genes of any sequenced diatom, with a large number of tandem duplicates [31]. *S. robusta* is a motile benthic species belonging to raphid pennates, and it is proposed that adaptions to this environment may have led to this genomic expansion. Indeed, an expansion of the iGluR family in penaeid shrimp has been hypothesised to allow rapid locomotive responses to aid survival in a benthic environment [112]. However, other diatom species that also exhibit a benthic phase, such as *P. tricornutum*, do not show the same genomic expansion.

The Ca^2+^-signalling machinery in diatoms therefore exhibits similarities to aspects of the well-characterised signalling mechanisms in both plants and animals. For example, diatoms lack IP_3_Rs but they possess Ca_v_ and TRP channels that are central to many signalling processes in animals. Diatoms and plants are both photosynthetic and have large central vacuole(s) that likely perform similar roles in the storage of inorganic ions and organic solutes. However, diatoms also exhibit fast Na^+^/Ca^2+^-based action potentials, similar to those observed in excitable animal cells. These rapid Ca^2+^-dependent signalling mechanisms are linked to motility in unicellular organisms, although it is unclear why non-motile diatom species also possess a suite of ion channels that facilitate rapid action potential-based signalling. One explanation may be the dynamic environment in which diatoms reside. Diatoms must be resilient to extreme temperature and osmotic fluctuations, as well as sudden changes in nutrient availability. Even though large centric diatoms appear non-motile, they can rapidly adjust their cell density and change sinking speeds through the water column on timescales of seconds [113]. It is likely that these processes require mechanisms for rapid cell signalling.

Diatoms inhabit highly diverse environments, including benthic and pelagic environments in marine and freshwater ecosystems. Moreover, diatoms have a truly global distribution, spanning from the sea-ice of polar regions to mountain lakes in the tropics. The broad diversity of diatom Ca^2+^-signalling mechanisms likely contributes to their ecological success across these hugely diverse environments. As more diatom genomes become available, we will be able to address how these major environmental transitions, such as the colonisation of freshwater environments, have influenced the evolution of diatom signalling mechanisms.

## Figures and Tables

**Table 1:** TPC1 and TPCL channels identified in diatom genomes.

Species	Diatom Group	TPC1	TPCL
*Chaetoceros tenuissimus*	Centric	2	1
*Minidiscus variabilis*	Centric	-	-
*Thalassiosira pseudonana*	Centric	1	-
*Thalassiosira oceanica*	Centric	1	-
*Fragilaria crotonensis*	Araphid pennate	1	1
*Pseudo-Nitzschia multiseries*	Raphid pennate	1	1
*Fragilariopsis cylindrus*	Raphid pennate	1	2
*Nitzschia inconspicua*	Raphid pennate	1	2
*Nitzschia putrida*	Raphid pennate	1	2
*Phaeodactylum tricornutum*	Raphid pennate	1	1
*Seminavis robusta*	Raphid pennate	1	1
*Fistulifera solaris*	Raphid pennate	2	2
*Mayamaea pseudoterrestris*	Raphid pennate	-	1

## Data Availability

All of the sequence data used in this study were obtained from public repositories. Further manipulations of these data (e.g., multiple sequence alignments) are available from the authors on request.

## Supplementary Materials

The following supporting information can be downloaded at: https://www.mdpi.com/article/10.3390/plants13091207/s1, Data S1: OSCA alignment; Data S2: Orai alignment; Data S3: TPC alignment; Data S4: TRP alignment; Data S5: pPha-T1-Venus; Table S1: Protein Accessions. References [114,115,116,117,118,119,120,121,122,123,124,125] are cited in the supplementary materials.
