# Supplementary material for: Channels of Evolution: Unveiling Evolutionary Patterns in Diatom Ca2+ Signalling
Source: Plants (Basel). 2024 Apr 26;13(9):1207. doi: 10.3390/plants13091207 (PMC11085791; doi:10.3390/plants13091207)
Supplement: Supplementary file 1 [file plants-13-01207-s001.zip › Supplementary Data S5 pPha-T1-Venus.pdf]

Supplementary Data S5: Sequence information for pPha-T1-Venus vector

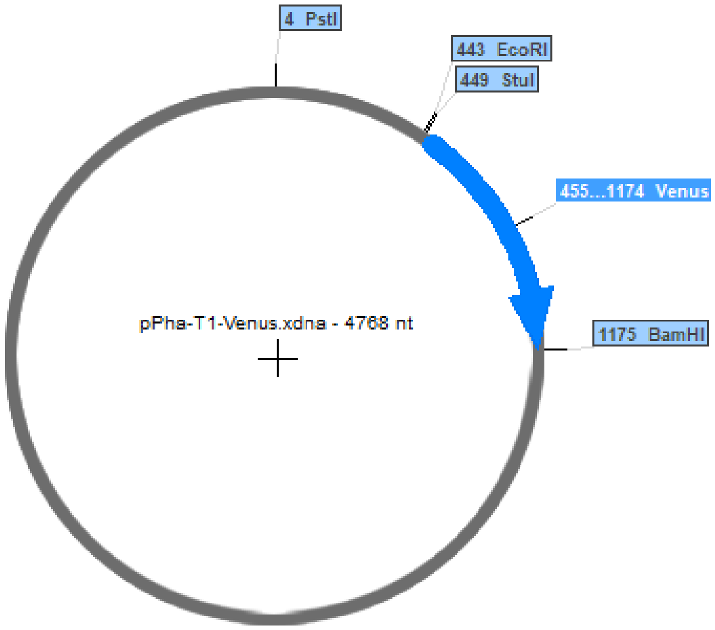

pPha-T1-Venus

A codon optimised Venus gene was synthesised and cloned into pPha-T1 via *EcoRI* and *BamHI*. Cutting with *PstI* and *StuI* removes the *fcpA* promoter, allowing the gene of interest (without stop codon) and its native promoter to be inserted in frame with Venus. This results in expression of the gene of interest with a C-terminal Venus fusion.

The entire sequence is shown below. *PstI* and *StuI* sites are underlined and the Venus coding sequence is shaded.

>pPha-T1-Venus

GGGCTGCAGGACGCAATGGAGGATTATCACCGCAAAAATGAACTTCGAAAAAAACTTTCGAGCGACCA
TGGAAAAGGAGGATCAGATTCAGATTACAACAGTGGATTGCTCTGGTAGCAAATATCTTCTGCTAGAT
TGGCTCATGGTCGGTTTTGGACGTTCGAAGCTCACCGTCAAAAGAAACAAAAGAGAAGAATGACGTCT
TCGTGACGTAGAATCTACGACTGTACTCGGATCTGGGAAATGAATTGACTCACGGTCTTCTTCGAGTC
CTGTTACAGGCCCTTGGTCCGAACCCCCACACGATTTTTGCACCAAAGATTTGCTTCAATTTGCTGGA
TGTTTTGACTGCAAGATCAGCTGGCCTAGCAAGAGTGCTCGTGTTGCTTCGTCGGGAATCCCTACGAA
TTTCAGTTCTGCACAAATTTGTCTGCCGTTTCGAGAATTCAGGCCTATGGTTTCCAAGGGAGAAGAAC
TCTTCACCGGTGTCGTCCCCATTCTCGTCGAACTCGACGGAGACGTCAACGGTCACAAGTTTTCCGTC
TCCGGCGAAGGAGAAGGTGACGCCACCTACGGCAAGCTCACCCTCAAGCTCATCTGCACCACCGGAAA
GTTGCCGGTCCCCTGGCCGACCTTGGTCACCACCTTGGGATACGGACTCCAGTGCTTCGCCCGCTACC
CCGACCACATGAAGCAGCACGACTTCTTTAAGTCCGCCATGCCCGAAGGATACGTCCAGGAACGTACC
ATTTTCTTTAAGGACGACGGTAACTACAAGACCCGCGCCGAAGTCAAGTTCGAAGGTGACACCCTCGT
CAACCGTATTGAACTCAAGGGCATCGACTTTAAGGAAGACGGCAACATCCTCGGACACAAGCTCGAAT
ACAACTACAACTCCCACAACGTCTACATTACCGCCGACAAGCAGAAGAACGGAATCAAGGCCAACTTC

AAGATTCGCCACAACATCGAAGACGGAGGTGTCCAGCTCGCCGACCACTACCAGCAGAACACCCCCGAT
TGGAGATGGACCGGTCCTCCTCCCGGACAACCACTACCTCTCCTACCAGTCCAAGCTCTCCAAGGACC
CCAACGAAAAGCGTGACCACATGGTCCTCCTCGAATTTGTCACCGCCGCCGGTATCACCTTGGGAATG
GACGAACTCTACAAGTGAGGATCCTCTAGAGCTTCAGAAGCGTGCTATCGAACTCAACCAGGGACGTG
CGGCACAAATGGGCATCCTTGCTCTCATGGTGCACGAACAGTTGGGAGTCTCTATCCTTCCTTAAAAA
TTTAATTTTCATTAGTTGCAGTCACTCCGCTTTGGTTTCACAGTCAGGAATAACACTAGCTCGTCTTC
ACCATGGATGCCAATCTCGCCTATTCATGGTGTATAAAAGTTCAACATCCAAAGCTAGAACTTTTGGA
AAGAGAAAGAATATCCGAATAGGGCACGGCGTGCCGTATTGTTGGAGTGGACTAGCAGAAAGTGAGGA
AGGCACAGGATGAGTTTTCTCGAGACATACCTTCAGCGTCGTCTTCACTGTCACAGTCAACTGACAGT
AATCGTTGATCCGGAGAGATTCAAAATTCAATCTGTTTGGACCTGGATAAGACACAAGAGCGACATCC
TGACATGAACGCCGTAAACAGCAAATCCTGGTTGAACACGTATCCTTTTGGGGGCCTCCGCTACGACG
CTCGCTCCAGCTGGGGCTTCCTTACTATACACAGCGCGCATATTTCACGGTTGCCAGATGTCAAGATG
GCCAAGTTGACCAGTGCCGTTCCGGTGCTCACCGCGCGCGACGTCGCCGGAGCGGTCGAGTTCTGGAC
CGACCGGCTCGGGTTCTCCCGGGACTTCGTGGAGGACGACTTCGCCGGTGTGGTCCGGGACGACGTGA
CCCTGTTCATCAGCGCGGTCCAGGACCAGGTGGTGCCGGACAACACCCTGGCCTGGGTGTGGGTGCGC
GGCCTGGACGAGCTGTACGCCGAGTGGTCGGAGGTCGTGTCCACGAACTTCCGGGACGCCTCCGGGCC
GGCCATGACCGAGATCGGCGAGCAGCCGTGGGGGCGGGAGTTCGCCCTGCGCGACCCGGCCGGCAACT
GCGTGCACTTCGTGGCCGAGGAGCAGGACTGAACCTTCCTTAAAAATTTAATTTTCATTAGTTGCAGT
CACTCCGCTTTGGTTTCACAGTCAGGAATAACACTAGCTCGTCTTCACCATGGATGCCAATCTCGCCT
ATTCATGGTGTATAAAAGTTCAACATCCAAAGCTAGAACTTTTGGAAAGAGAAAGAATATCCGAATAG
GGCACGGCGTGCCGTATTGTTGGAGTGGACTAGCAGAAAGTGAGGAAGGCACAGGATGAGTTTTCTCG
AGGCCGGTCTCCCTATAGTGAGTCGTATTAATTTCGATAAGCCAGGTTAACCTGCATTAATGAATCGG
CCAACGCGCGGGGAGAGGCGGTTTGCGTATTGGGCGCTCTTCCGCTTCCTCGCTCACTGACTCGCTGC
GCTCGGTCGTTCGGCTGCGGCGAGCGGTATCAGCTCACTCAAAGGCGGTAATACGGTTATCCACAGAA
TCAGGGGATAACGCAGGAAAGAACATGTGAGCAAAAGGCCAGCAAAAGGCCAGGAACCGTAAAAAGGC
CGCGTTGCTGGCGTTTTTCCATAGGCTCCGCCCCCCTGACGAGCATCACAAAAATCGACGCTCAAGTC
AGAGGTGGCGAAACCCGACAGGACTATAAAGATACCAGGCGTTTCCCCCTGGAAGCTCCCTCGTGCGC
TCTCCTGTTCCGACCCTGCCGCTTACCGGATACCTGTCCGCCTTTCTCCCTTCGGGAAGCGTGGCGCT
TTCTCAATGCTCACGCTGTAGGTATCTCAGTTCGGTGTAGGTCGTTCGCTCCAAGCTGGGCTGTGTGC
ACGAACCCCCCGTTCAGCCCGACCGCTGCGCCTTATCCGGTAACTATCGTCTTGAGTCCAACCCGGTA
AGACACGACTTATCGCCACTGGCAGCAGCCACTGGTAACAGGATTAGCAGAGCGAGGTATGTAGGCGG
TGCTACAGAGTTCTTGAAGTGGTGGCCTAACTACGGCTACACTAGAAGGACAGTATTTGGTATCTGCG
CTCTGCTGAAGCCAGTTACCTTCGGAAAAAGAGTTGGTAGCTCTTGATCCGGCAAACAAACCACCGCT
GGTAGCGGTGGTTTTTTTGTTTGCAAGCAGCAGATTACGCGCAGAAAAAAAGGATCTCAAGAAGATCC
TTTGATCTTTTCTACGGGGTCTGACGCTCAGTGGAACGAAAACTCACGTTAAGGGATTTTGGTCATGA
GATTATCAAAAAGGATCTTCACCTAGATCCTTTTAAATTAAAAATGAAGTTTTAAATCAATCTAAAGT
ATATATGAGTAAACTTGGTCTGACAGTTACCAATGCTTAATCAGTGAGGCACCTATCTCAGCGATCTG
TCTATTTCGTTCATCCATAGTTGCCTGACTCCCCGTCGTGTAGATAACTACGATACGGGAGGGCTTAC
CATCTGGCCCCAGTGCTGCAATGATACCGCGAGACCCACGCTCACCGGCTCCAGATTTATCAGCAATA
AACCAGCCAGCCGGAAGGGCCGAGCGCAGAAGTGGTCCTGCAACTTTATCCGCCTCCATCCAGTCTAT
TAATTGTTGCCGGGAAGCTAGAGTAAGTAGTTCGCCAGTTAATAGTTTGCGCAACGTTGTTGCCATTG
CTACAGGCATCGTGGTGTCACGCTCGTCGTTTGGTATGGCTTCATTCAGCTCCGGTTCCCAACGATCA
AGGCGAGTTACATGATCCCCCATGTTGTGCAAAAAAGCGGTTAGCTCCTTCGGTCCTCCGATCGTTGT
CAGAAGTAAGTTGGCCGCAGTGTTATCACTCATGGTTATGGCAGCACTGCATAATTCTCTTACTGTCA
TGCCATCCGTAAGATGCTTTTCTGTGACTGGTGAGTACTCAACCAAGTCATTCTGAGAATAGTGTATG
CGGCGACCGAGTTGCTCTTGCCCGGCGTCAATACGGGATAATACCGCGCCACATAGCAGAACTTTAAA
AGTGCTCATCATTGGAAAACGTTCTTCGGGGCGAAAACTCTCAAGGATCTTACCGCTGTTGAGATCCA
GTTCGATGTAACCCACTCGTGCACCCAACTGATCTTCAGCATCTTTTACTTTCACCAGCGTTTCTGGG
TGAGCAAAAACAGGAAGGCAAAATGCCGCAAAAAAGGGAATAAGGGCGACACGGAAATGTTGAATACT
CATACTCTTCCTTTTTCAATATTATTGAAGCATTTATCAGGGTTATTGTCTCATGAGCGGATACATAT
TTGAATGTATTTAGAAAAATAAACAAATAGGGGTTCCGCGCACATTTCCCCGAAAAGTGCCACCTGAC

GTCTAAGAAACCATTATTATCATGACATTAACCTATAAAAATAGGCGTATCACGAGGCCCTTTCGTCT
CGCGCGTTTCGGTGATGACGGTGAAAACCTCTGACACATGCAGCTCCCGGAGACGGTCACAGCTTGTC
TGTAAGCGGATGCCGGGAGCAGACAAGCCCGTCAGGGCGCGTCAGCGGGTGTTGGCGGGTGTCGGGGC
TGGCTTAACTATGCGGCATCAGAGCAGATTGTACTGAGAGTGCACCATATGGACATATTGTCGTTAGA
ACGCGGCTACAATTAATACATAACCTTATGTATCATACACATACGATTTAGGTGACACTATAGAACCA
GATCCCCC
